# Pedestrian flow characteristics through different angled bends: Exploring the spatial variation of velocity

**DOI:** 10.1371/journal.pone.0264635

**Published:** 2022-03-03

**Authors:** Jamal Hannun, Charitha Dias, Alaa Hasan Taha, Abdulaziz Almutairi, Wael Alhajyaseen, Majid Sarvi, Salim Al-Bosta

**Affiliations:** 1 Department of Civil and Architectural Engineering, Qatar University, Doha, Qatar; 2 Qatar Transportation and Traffic Safety Center, College of Engineering, Qatar University, Doha, Qatar; 3 Department of Infrastructure Engineering, The University of Melbourne, Melbourne, VIC, Australia; 4 Supreme Committee for Delivery and Legacy, Al Bidda Tower, Corniche Street, Doha, Qatar; University of Shanghai for Science and Technology, CHINA

## Abstract

Common geometrical layouts could potentially be bottlenecks, particularly during emergency and high density situations. When pedestrians are interacting with such complex geometrical settings, the congestion effect might not be uniform over the bottleneck area. This study uses the trajectory data collected through a controlled laboratory experiment to explore the spatial variation of speeds when a group of people navigates through bends. Four turning angles, i.e., 45°, 90°, 135° and 180°, with a straight corridor and two speed levels, i.e., normal speed walking and slow running (jogging), were considered in these experiments. Results explained that the speeds are significantly different over the space within the bend for all angles (except 0°) under both speed levels. In particular, average walking speeds are significantly lower near the inner corner of the bend as compared to the outer corner. Further, such speed variations are magnified when the angle of the bend and desired speed increase. These outcomes indicate that even smaller turning angles, e.g., 45° could create bottlenecks near the inner corner of the bend, particularly when the walking speeds are high. The findings of this study could be useful in understanding the congestion and bottleneck effects associated with complex geometrical settings, and calibrating microscopic simulation tools to accurately reproduce such effects.

## 1. Introduction

Major public infrastructures are heavily used by large crowds not only during special events but also during daily peak hours for regular commuting purposes. Under emergency situations, such mass gatherings could result in stampedes and fatal accidents [1, 2]. For example, during the annual Hajj pilgrimage in Saudi Arabia back in 2015, over 2000 fatalities were reported due to a serious stampede accident. Moreover, 21 fatalities and over 500 injuries were reported during the Love Parade accident that occurred in Duisburg, Germany in 2010. Furthermore, in 2014, 36 fatalities were reported in Shanghai due to a stampede during the New Year celebration. The considerable detrimental social impacts that arise from stampede accidents have led the researchers to draw significant attention to investigate the dynamics of crowds and their safety in the last decade [3, 4].

In general, the movement of large crowds could be significantly affected by their interactions with the geometrical settings of the walking space. This in turn could create bottlenecks and cause stampedes. The formation of congestion could be considerably different across different architectural and geometrical settings [5]. Hence, the design of public crowd gathering spaces to withstand large crowds, while ensuring safety and efficiency, is a challenging task. In order to design crowd gathering places for various purposes, such as daily commuting, special events, mega-events, and different situations, e.g., normal circulations, emergency evacuations, it is important to have a proper understanding of the flow of the crowds. In particular, understanding the interactions of crowds with different complex geometrical features under different conditions is vital. Such understanding is crucial in locating bottlenecks and to optimizing geometrical settings that warrant an efficient and safe movement of pedestrian crowds. This is because, on a microscopic level, pedestrian behaviors associated with various geometrical settings in the walking space tend to considerably differ from one condition to another. Moreover, having this understanding will aid in managing large crowds and predicting their behavior in public spaces.

In recent years, microscopic pedestrian simulation tools that are based on different behavior models have been adopted to predict bottlenecks and hazardous crowd movements [6]. Such crowd simulation tools can be used in the design of public spaces [7] as well as in policy making purposes [8]. It is mandatory to assess the reliability of such pedestrian models by calibrating and validating them with experimental data collected under diverse conditions [9, 10]. Empirical data used for calibrating and validating the models of pedestrian behavior can be either collected through actual field observations or controlled experiments. Even though the provision of bias-free data makes actual field observations advantageous over lab experiments, in real-world field observations, it is difficult to detect a particular behavior for several replicates within the same investigated parameters. That is, isolating the parameter that is being investigated by controlling other parameters is difficult. Therefore, collecting data through controlled laboratory experiments is more feasible for research studies. Nevertheless, the availability of valid and reliable experimental data is of vital importance for understanding the behavior of crowds, their interactions with the surrounding geometrical features, and for assessing the reliability and validity of crowd simulation models [11].

This study uses trajectory data collected through a controlled experiment to investigate the combined effect of turning angle and desired speed on pedestrians’ walking behaviors through bends. The key objective of this study is to understand and quantify the spatial variation of crowd flow characteristics through different angled bends under two desired speed levels. Instead of examining the general effects of turning angle and desired speed, the variations in velocity and density within and in the vicinity of the bottleneck area, i.e., near the bend, are analyzed in detail to locate the pinch point within the bottleneck. Such detailed examination will be helpful in understanding the mechanism of the occurrence of the congestion associated with bends and in devising design solutions.

This paper is structured as follows: In Section 2, a comprehensive literature review about the studies on the pedestrian behavior is provided. Then, in Section 3, the data collection and extraction procedures are explained in detail. This is followed by Section 4 that presents a discussion on the data and results. Finally, the discussion and conclusion of the study are presented in Section 5.

## 2. Related works

Numerous studies have investigated the different aspects of crowd dynamics using empirical methods [5, 12]. Different geometrical and architectural configurations with different level of complexities, e.g., exits and entrances [13–17], straight corridors [18–20], crossing [21–25], merging [26–30], and turning [31–34] configurations, have been explored in such previous studies using controlled experiments conducted with human subjects. Not only humans, but non-human biological entities, e.g., ants, have also been used to explore the evacuation performance of different geometrical settings under panic conditions [35–37]. For the design purposes that ensure the safety of crowds and efficiency of crowd flows at general bottleneck scenarios, e.g., exit doors and corridors, several studies have indicated that there is a direct relationship between pedestrian flow and bottleneck width, i.e., increasing pedestrian flows with increasing the bottleneck width and thereby decrease in evacuation time [17, 38–41]. The outcomes of Liao et al. [17] indicated that the speed and density within the bottleneck were independent of bottleneck width, however, those before the bottleneck were dependent on the exit width. Through an experimental study, Wang et al. [38] revealed that bottlenecks located at the middle of a wall resulted in higher flows and less evacuation times when compared to bottlenecks located at the corners. The study by Shi et al. [42] indicated that bottlenecks located right at the corner with a circular column of 60 cm in diameter distanced at 1 m from an exit showed considerably better performance than middle exits with free of obstacles at both normal walking and jogging conditions. This improvement was attributed to the stabilization of pedestrian lanes facilitated by the existence of a circular obstacle near the bottleneck. Helbing et al. [43] also experimentally verified that the clogging effect in front of an exit when people exit in a pushy way could be significantly reduced by placing an obstacle, e.g., a partition board. Frank and Dorso [44] presented numerical simulations using the social force model to explore the presence of obstacles during the evacuation process of pedestrians. They found that by placing an obstacle, e.g., a panel or board, in front the exit, the evacuation time can be reduced when compared to an obstacle-free exit. However, when the panel-like obstacle is placed far away from the exit, e.g., at twice of the exit width, the effect became non-significant indicating that the location of the obstacle is important.

Studies on pedestrian behavior through corridors have also been well studied in the literature. For example, Zhang et al. [26] conducted an experimental study with 350 participants for different corridor widths of 1.8 m, 2.4 m, and 3 m and indicated that for the same facility type, the fundamental diagrams for different widths can be merged into one diagram when the specific flow (flow divided by the width) is used. However, when the flow is bidirectional, the capacity (maximum specific flow) of the corridor tends to decrease [32]. The formation of self-organized lanes improves the flow through corridors and Seyfried et al. [19] explained that for unidirectional flows the lane-formation phenomena can be observed when the corridor widths exceed 0.9 m.

For crossing scenarios, Wong et al. [22] conducted an experiment considering 4 crossing angles, i.e., 45°, 90°, 135°, and 180°, and concluded that by increasing the intersecting angle, the operational capacity of intersections tends to reduce. On the contrary, Zhang and Seyfried [45] indicated that there is no significant difference between the fundamental diagrams of 90° and 180° intersecting angles. Aghabayk et al. [24] considered three crossing angles (30°, 90, and 150°) and two speed levels (normal speed walking and jogging) and concluded that crossing configurations with smaller angles (30° in their experiment) have higher flow rates and smaller evacuation times. Lian et al. [25] conducted an experiment to study four-directional intersecting flows with 364 students. Their results indicated that, during extremely crowded situations, the local density in the middle of the crossing area (or at the intersection) could exceed 10 ped/m^2^, however, the densities in the corridors would be considerably lower as compared to the intersection.

Regarding merging scenarios, Zhang et al. [26] compared the fundamental diagrams for straight corridors and T-junctions. The comparisons indicated that the fundamental diagrams for the region after the merging of the two streams do not deviate from the fundamental diagrams for unidirectional pedestrian flows through a straight corridor. However, before merging as the speeds are significantly lower due to the change in the geometry, the fundamental diagrams tend to deviate from those for straight corridors. Moreover, Shiwakoti et al. [28] studied the influence of merging angle on pedestrians flow characteristics. They considered three merging angles, i.e., 60°, 90°, and 180° and found that the average speed within, before and after the merging area decreases with increasing merging angle. Additionally, Shahhoseini and Sarvi [46] revealed that asymmetrical merging layouts tend to be less efficient in terms of evacuation time, speed, and discharge rate compared to the equivalent symmetric layouts.

Zhang et al. [32] compared the fundamental diagrams for 90° corners and T-junctions and concluded that the fundamental diagrams for before and after the corner are well-matched with those for after merging. However, the fundamental diagrams before the merging considerably deviated from the above three cases. They further specified that higher density regions emerge close to the junction. Dias et al. [31] studies pedestrian behaviors through 5 different angled bends (45°, 60°, 90°, 135°, and 180°) under solo walking conditions and concluded that the speed is reduced with increasing turning angle. Considering three speed levels, i.e., normal speed walking, fast speed walking and slow speed running, they further verified that the percentage speed reduction is magnified when the desired speed increases. Uni- and bi-directional flows at a 90° bend were considered in the controlled experiment conducted by Ye et al. [33]. They concluded that the velocities for the pedestrians walking at outer lanes tend to be higher. Further, the angular speeds for the pedestrians walking at inner lanes tend to be higher as compared to the outer lanes.

According to the conducted literature review, it can be noted that a wide range of geometrical settings in walking spaces, which can potentially be bottlenecks under different walking conditions, have been considered in previous studies. In general, such studies have examined the bottleneck effects and capacities of such different geometrical layouts considering the bottleneck as one unit. Even though very few studies, e.g., [25, 32, 33], presented the distributions of speeds and densities within the bottleneck area, detailed quantitative evaluations have not been presented. Detailed microscopic examinations of the characteristics of velocity and density distributions over space and time could be useful in identifying the bottleneck effect and the mechanism of the occurrence the congestion, particularly when pedestrian crowds interact with complex geometrical settings. Considering such gaps in the knowledge, this study examines the distributions of velocities and densities over the walking space of angled corridors, particularly in the vicinity of the bend which is the most critical location, under two desired speed levels.

## 3. Methods

### 3.1. Controlled experiments

To understand the pedestrians’ behavior when they negotiate bends, a controlled experiment was carried out at Monash University, Australia. In total, 55 people (30 males and 25 females) agreed to participate in these human experiments with written consent. The participants were mainly the students and staff of Monash University. That is, walking behavior of a young crowd is represented in these experiments. The experiment was conducted following the guidelines stipulated by the Human Research Ethics Committee of Monash University. Four turning angles, i.e., 45°, 90°, 135°, and 180°, and a straight corridor (0° turning), were considered. Further, a constant corridor width of 1.5 m was considered.

**Fig 1:**
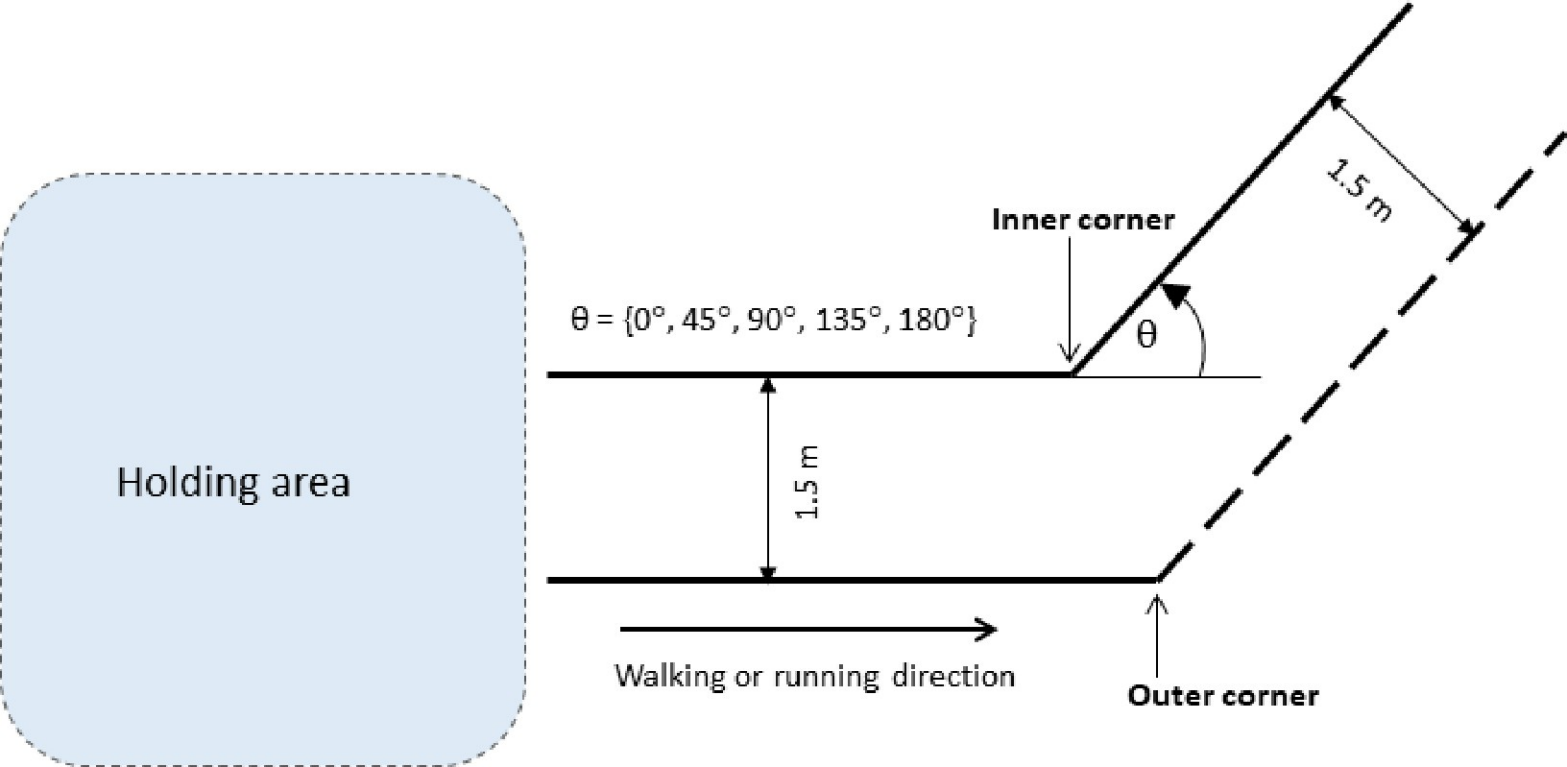
Schematic representation of the experiment setup.

Two speed levels were considered to represent normal speed walking and slow speed running (jogging) conditions. Under these experimental conditions, the estimated average speeds through straight corridors were 1.07 m/s and 2.14 m/s under normal speed walking and jogging conditions, respectively [47]. Corresponding free-flow speeds for normal speed walking and jogging were 1.44 m/s and 3.01 m/s, respectively [31]. A schematic representation of the experiment setup is shown in Fig 1.

The purpose of the experiments was not revealed to the participants. The participants of this experiment were first instructed to gather inside the holding area (approximately 6 m x 6 m space). For walking experiments, they were asked to walk through the different settings of the corridors at normal walking speed and for jogging experiments, they were asked to slowly run as if they jog through the configurations. Only unidirectional (one-way) scenarios were considered in this experiment. That is, participants walked or ran in the direction indicated in Fig 1. To reduce the influence of pedestrians’ boredom and fatigue, breaks were provided on a regular basis throughout the experiments. A digital video camera mounted at a higher elevation was used to record the experiments.

### 3.2. Data extraction and analysis

The coordinates of the participants were retrieved via an image sequence obtained from the recordings of the digital video camera. That is, the positions of each pedestrian were manually extracted at 0.12 s time intervals. The participants’ trajectories were then obtained by converting the extracted image coordinates into ground coordinates by using the two-dimensional projective transformation method [48]. Extracted trajectories are presented in S1 Appendix. For the analyses, first and last few trajectories (around 10 trajectories) were removed to consider only the non-free flow trajectories.

MATLAB software was used to generate speed heat maps in terms of (x,y,v), where (x,y) is the location and v is the instantaneous speed of individual pedestrians, for all the angles and both speed levels. These heat maps were used to qualitatively and quantitatively evaluate the speed distributions over the space within the turning corridor. Moreover, velocity vector maps were also generated using MATLAB in terms of (x, y, vx, vy), where, vx and vy are the x and y component of the velocity, respectively, to describe the velocity vector distributions and the change in directions. These velocity vectors illustrate the pedestrians’ turning velocity along the lateral and longitudinal direction of the corridor. In addition, density heat maps were created considering occupancies to show the critical locations where stampedes could occur. The effect of initial speed and bend angle is illustrated by showing the spatial distribution of density throughout the corridors. The generated heat maps for longitudinal velocity and density were created by assigning the experimental coordinates to a plane with a mesh size of 10 cm x10 cm, covering the full space of the corridor, these measures were smoothed using a convolution with a Gaussian function as pedestrians characteristics tend to vary in a Gaussian distribution thought the corridor [49, 50].

The velocities were compared throughout 2 m before and after the inner corner of the bend. To examine the effect of the location in the bends on the velocity of pedestrians, the 1.5 m corridor was divided into 0.5 m lanes to represent inner (close to the inner corner of the bend), middle, and outer (close to the outer corner of the bend) lanes. Further, the bend segment of the corridor, i.e., 2 m before and after the inner corner, was divided into five portions of 0.8 m length along the longitudinal direction.

To compare the average speeds at the bend (within a range of 0.4 m before and after the inner corner and outer corner) between different angled corridors for each speed level, the t-test was performed. To compare the average speeds within the bend at different locations (at the inner, middle, and outer lanes) the ANOVA tests were performed for each angle and speed level.

## 4. Results

### 4.1. Spatial density distributions

**Fig 2:**
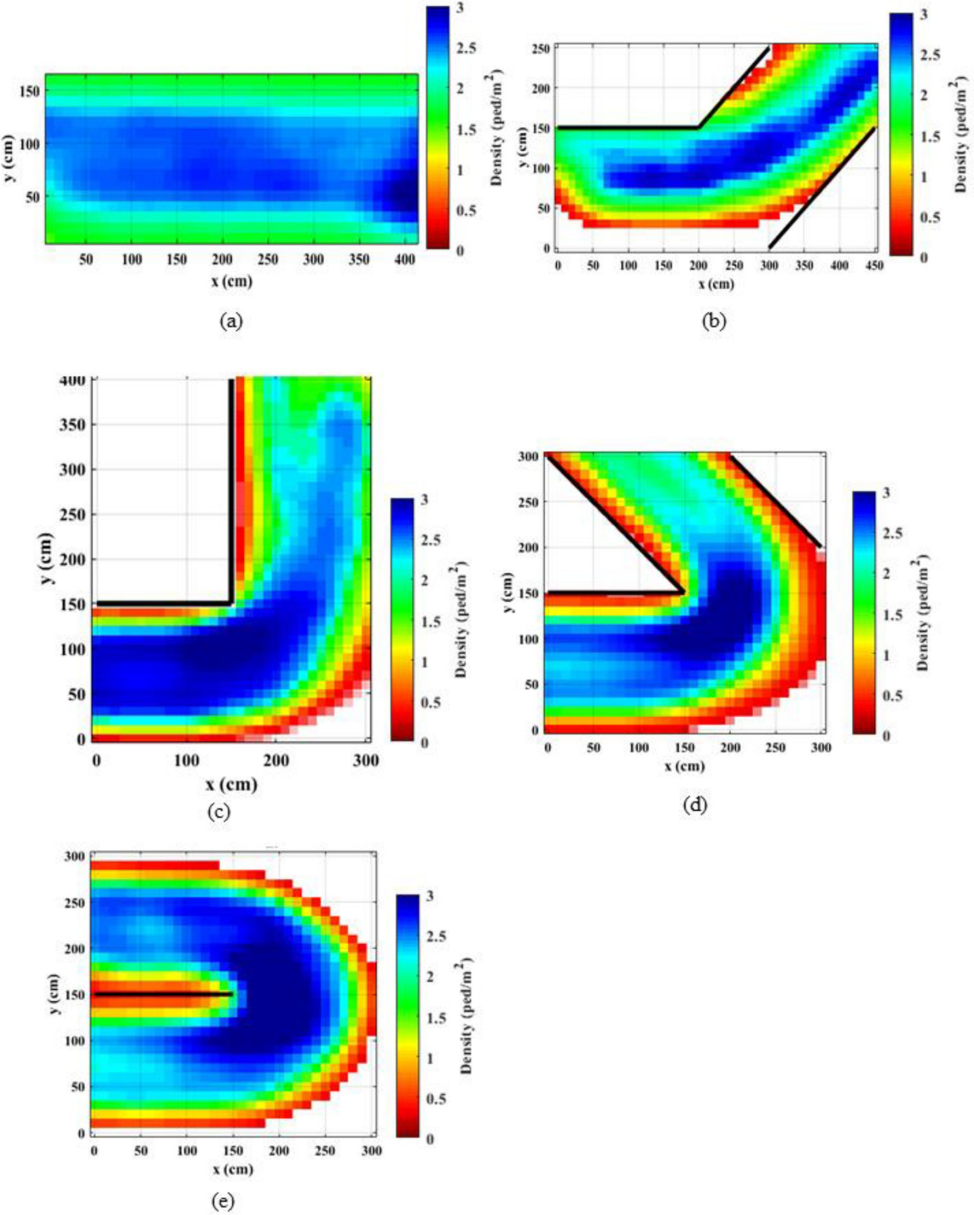
Density heat maps for normal speed walking; (a) straight corridor (0°); (b) 45°; (c) 90°; (d) 135°; (e) 180°.

**Fig 3:**
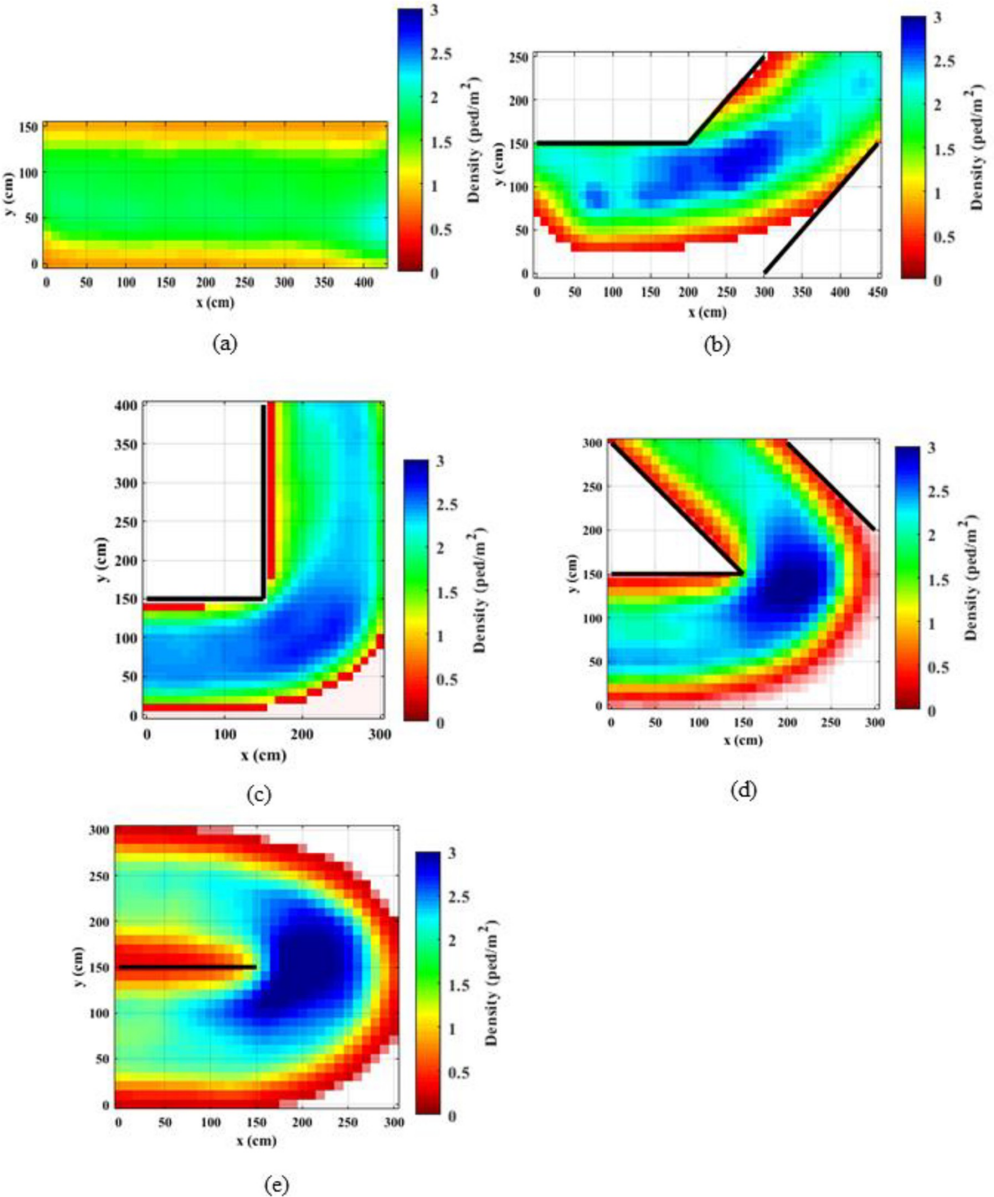
Density heat maps for slow speed running; (a) straight corridor (0°); (b) 45°; (c) 90°; (d) 135°; (e) 180°.

Density distributions throughout the corridors were calculated using the occupancies of the meshes of 10 cm x10 cm size. The presence of pedestrians inside these meshes was recorded and averaged throughout the experiment time. Figs 2 and 3 present the spatial distribution of the densities for the normal walking speed and jogging cases, respectively. It is observed that the densities reach up to 3 ped/m^2^, particularly near the bend. It can be noted that for straight corridors, the density distributions are consistent throughout the corridor both walking and jogging cases. Further, for the straight corridors and the straight portion of the angled corridors, the densities of the normal speed walking cases are higher as compared to the jogging cases. When the speeds are higher, people tend to keep longer inter-personal distances [47] and as a result, the density becomes lower. For angled corridors, the densities considerably vary over the corridor and the densities are higher in the middle of the corridor compared to the edges (or near the walls) as people keep a safe distance from the walls. Observing density distributions in Fig 2 for the bends that are larger than 90°, it can be noted that the densities are higher in the vicinity of the bend compared to the start and the end of the corridor. The density distribution for the 90° normal speed case is qualitatively comparable with density distributions presented in Ye et al. [33]. However, in this study the maximum density at the bend reaches up to 3 ped/m^2^, whereas in Ye et al. [33] the maximum density is 2.5 ped/m^2^. Further, the clogging effect and the shockwave before the turning point is quite clear in current study.

Previous studies explained that exit flow rates are increased when the desired speed is increased, e.g., from normal speed walking to slow sped running [29, 47]. That means that the overall performance of the system could be enhanced by increasing the desired speed, e.g., by asking people to evacuate faster. However, as the findings of this study suggest, bottleneck effects could be triggered locally when the desired speeds are increased.

Comparing density distributions in Figs 2 and 3, it can be noted that the densities at the bend increase with the increasing turning angle. This observation indicates that the bottleneck effect becomes higher when the turning angle increases. Comparing density distributions for the same turning angle and different speed levels, it can be noted that for jogging cases the increase in the density is sudden. This effect is magnified when the turning angle is higher. This is mainly due to the sudden speed drop at the bend. These observations implies that higher turning angles at bends have a higher probability of causing stampedes, particularly when people are moving (or evacuating) faster.

### 4.2. Spatial distribution of speeds

**Fig 4:**
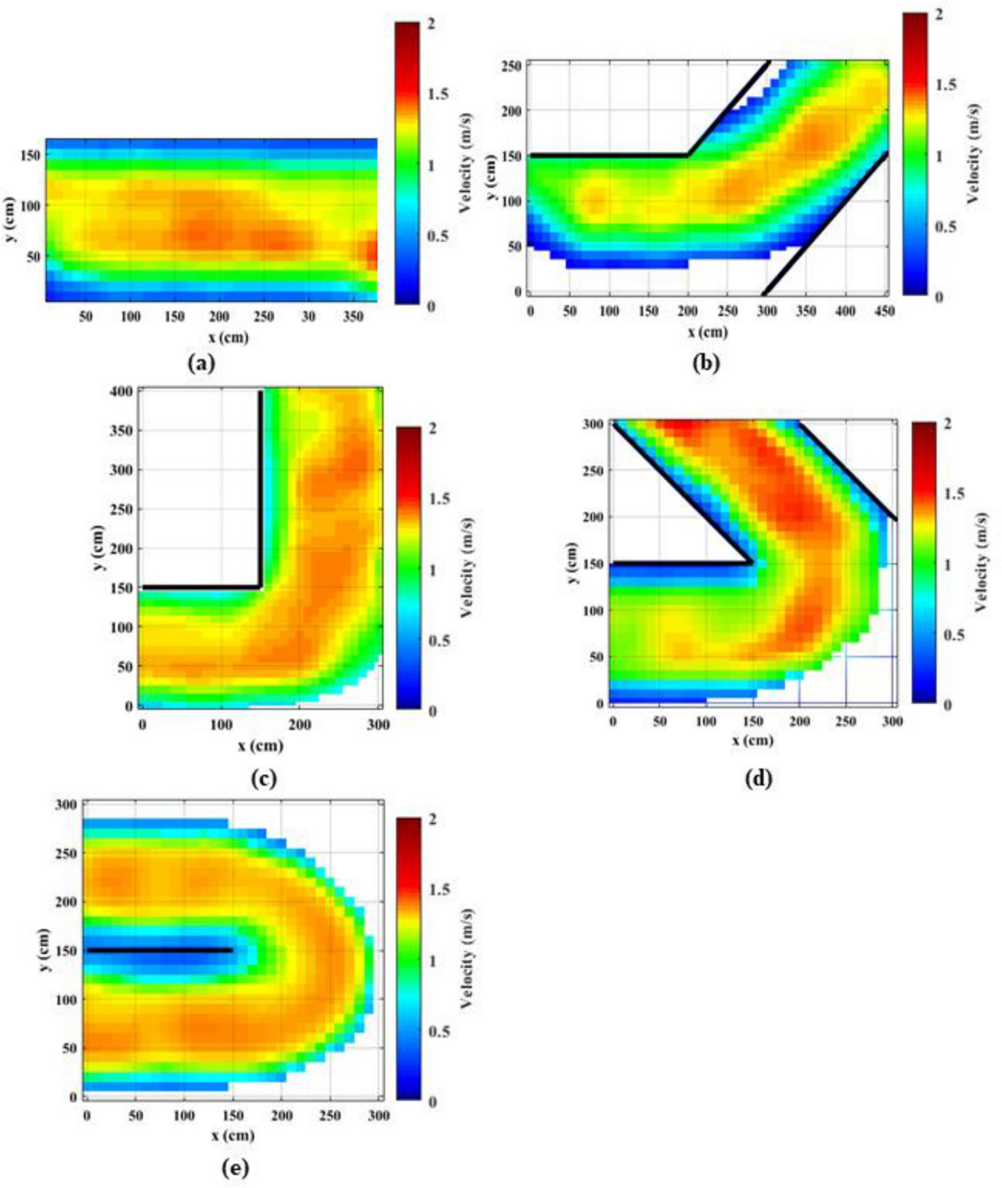
Distribution of speeds along the corridors for normal speed walking cases; (a) straight corridor (0°); (b) 45°; (c) 90°; (d) 135°; (e) 180°.

**Fig 5:**
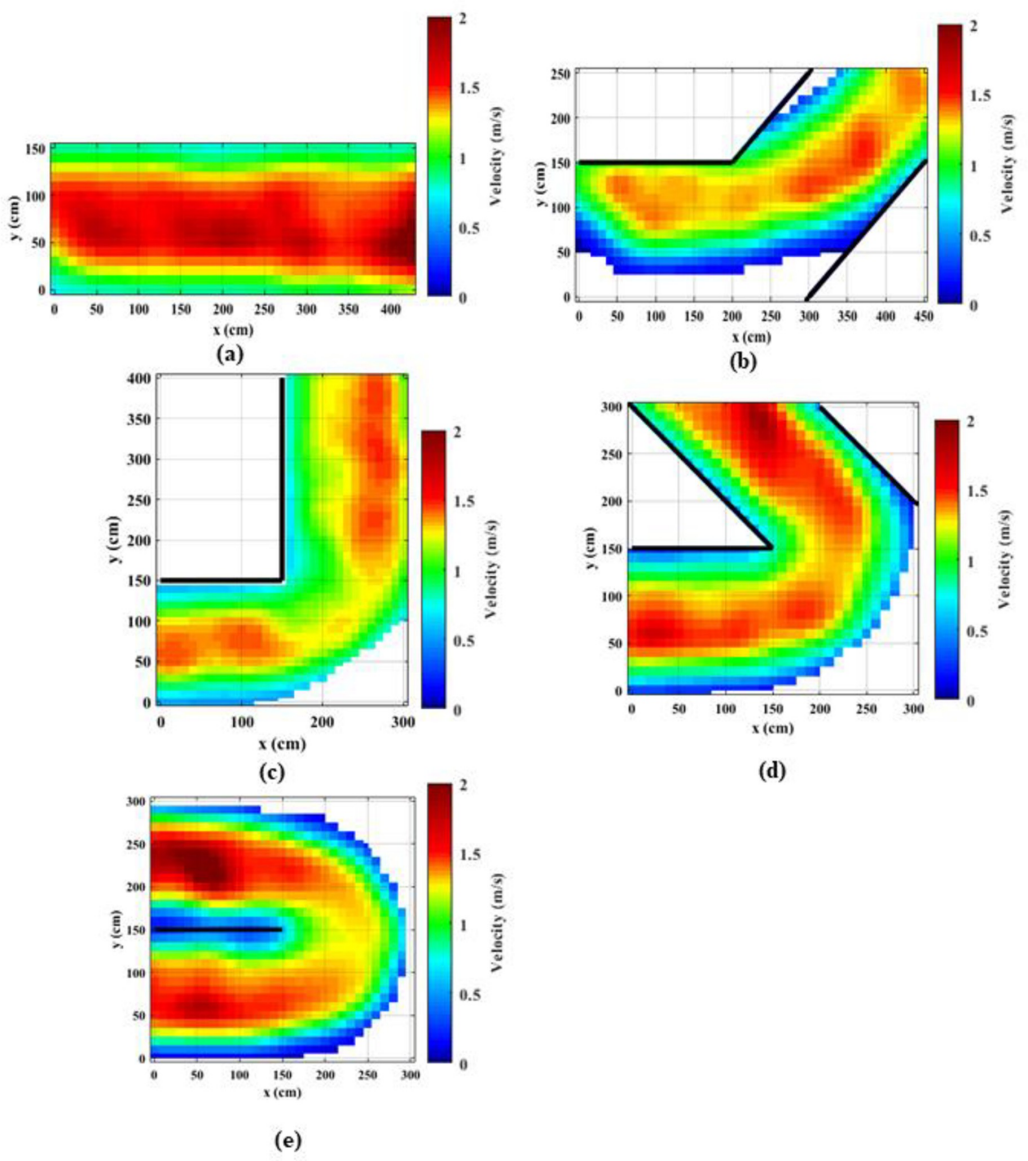
Distribution of speeds along the corridors for slow speed running (jogging) cases; (a) straight corridor (0°); (b) 45°; (c) 90°; (d) 135°; (e) 180°.

The instantaneous velocities (magnitudes and the directions) were estimated using the collected trajectory data (x, y, t) for each individual. The generated speed maps for normal speed walking and jogging cases are shown in Figs 4 and 5, respectively.

Observing these figures, it can be noted that speeds are not uniform over space. Because of the averaging and smoothing effect, speed reductions are not clear in the walking case (Fig 4). Nevertheless, it can be noted that the speeds near the inner corner are lower which is generally consistent with the findings of [33]. Observing Figs 4 and 5, it can be noted that the speeds are lower at the bend compared to approaching and receding areas. This reduction is magnified when the turning angle is increased. Observing higher tuning angles (Figs 4C–4E, and 5C–5E) it can be observed that the reduction in speed is higher near the inner corner of the bend. People have to traverse a larger radius near the outer corner of the bend compared to the inner corner and as a result, they have to reduce the speed at the inner corner compared to the outer corner. In addition, it can be noted that for the straight corridor case the speed distribution along the corridor is rather consistent.

The t-tests were conducted to compare the average speeds between different angled bends (within a range of 0.4 m before and after the inner corner and outer corner for each angle) and the outcomes for normal speed walking and jogging cases are summarized in Tables 1 and 2, respectively.

**Table 1:** t-test outcomes (t-stat, p-value) for the comparison of average speeds at the bend between different angled corridors for normal speed walking.

	0°	45°	90°	135°	180°
0°		(0.392, 0.348)	(13.313, <0.000)*	(19.055, <0.000)*	(23.809, <0.000)*
45°			(12.762, <0.000)*	(18.958, <0.000)*	(22.472, <0.000)*
90°				(8.175, <0.000)*	(10.899, <0.000)*
135°					(0.788, 0.215)
180°					

* Significant at 0.01 level.

**Table 2:** t-test outcomes (t-stat, p-value) for the comparison of average speeds at the bend between different angled corridors for jogging.

	0°	45°	90°	135°	180°
0°		(8.105, <0.000)*	(25.638,<0.000)*	(33.697, <0.000)*	(32.143,<0.000)*
45°			(14.481,<0.000)*	(21.711,<0.000)*	(19.676,<0.000)*
90°				(8.823, <0.000)*	(8.661,<0.000)*
135°					(0.393, 0.347)
180°					

* Significant at 0.01 level.

It can be noted that for normal speed walking, the difference between average speeds at the bend for the straight (0°) corridor and 45° corridor were statistically not significant. This implies that, for normal walking speeds and congested situations (maximum density = 3 ped/ m^2^), the turning angles that are less than 45° have no significant impact on the longitudinal walking speeds. The difference of the average speeds between 135° and 180° were not statistically significant which indicates that the influence of the angles beyond 135° is the same. For other angle combinations, the differences between the average speeds at the bend were statistically significant. For jogging cases, the differences between average speeds for different angle combinations were statistically significant except for the 135° and 180° combination (Table 2). This observation implies that for the angles beyond 135°, the effect of the turning angle has a similar effect on the collective speeds. Further, based on the statistics in Tables 1 and 2, it can be stated that when the desired speed increases (from normal speed walking to slow speed running) even smaller angles (e.g., 45°) could become bottlenecks y reducing the speeds significantly.

### 4.3. Spatial directional velocity vector maps

**Fig 6:**
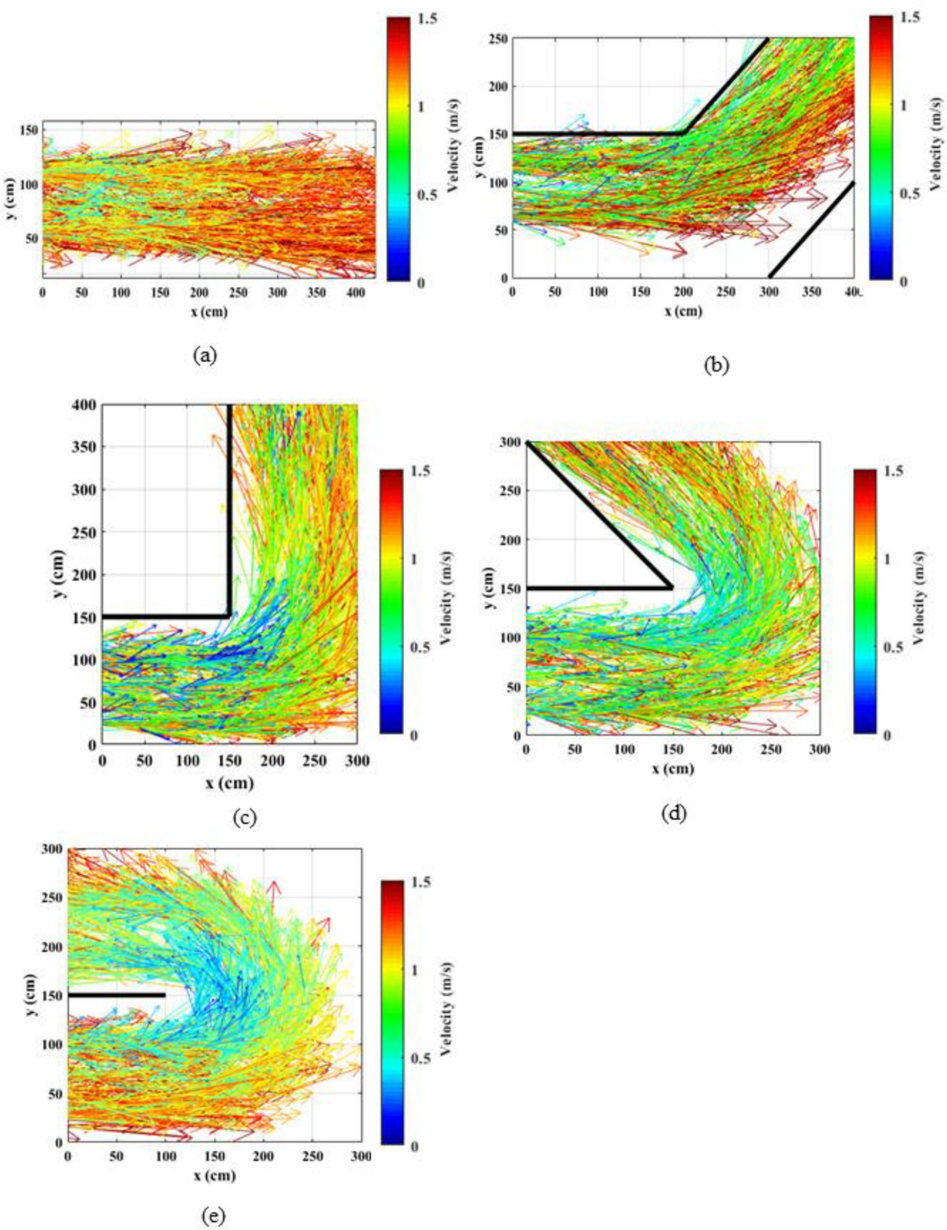
Distribution of velocity vectors along the corridors for normal speed walking through; (a) straight corridor (0°); (b) 45°; (c) 90°; (d) 135°; (e) 180° corridors.

**Fig 7:**
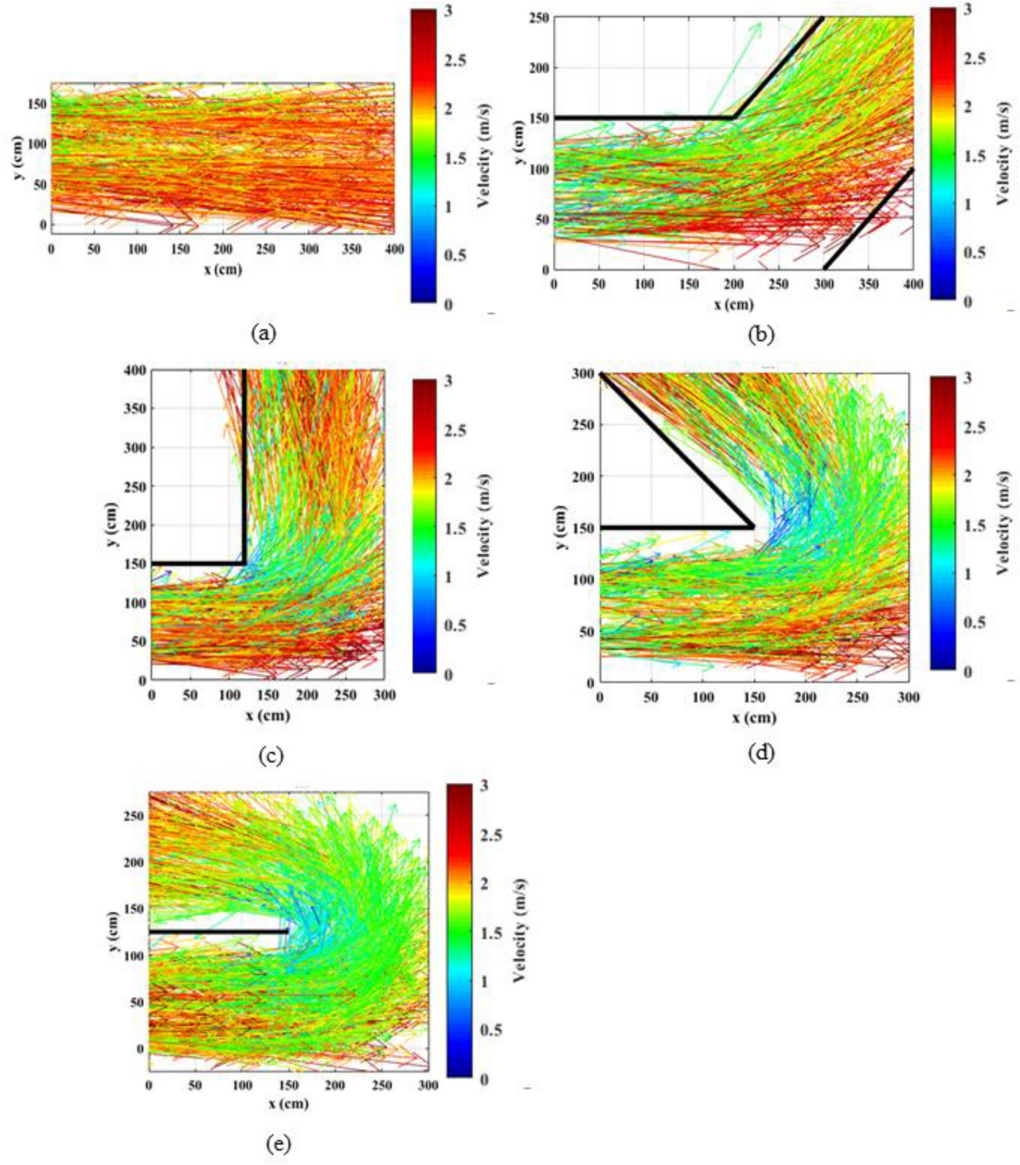
Distribution of velocity vectors along the corridors for slow speed running through; (a) straight corridor (0°); (b) 45°; (c) 90°; (d) 135°; (e) 180° corridors.

The instantaneous velocities (vx, vy) of pedestrians were estimated using the coordinates of individual pedestrians. Based on the estimated velocity vectors (vx, vy), vector maps were generated to show the variation in speed through the corridor area. Velocity vector maps for normal speed walking and jogging cases are shown in Figs 6 and 7, respectively. The arrow colors illustrate the magnitudes of the velocities while the directions of the arrows show the instantaneous directions of the velocities. From the vector maps, it can be visualized that the magnitudes of the speeds are lower near the inner corner of the bend compared to the outer corner and the velocity magnitudes increase toward the outer corner of the corridor. Variations in velocity magnitudes are not prominent in straight corridors and straight portions of the angled corridors that are far from the bend. It can further be noted that for both speed levels, the variations in speeds at the bend are magnified with the increase in the turning angle. In order to quantify these effects in detail and to statistically compare the velocity magnitudes at different locations of the bend, the corridors were divided into several regions as discussed in the next section.

### 4.4. Longitudinal velocities distributions

**Fig 8:**
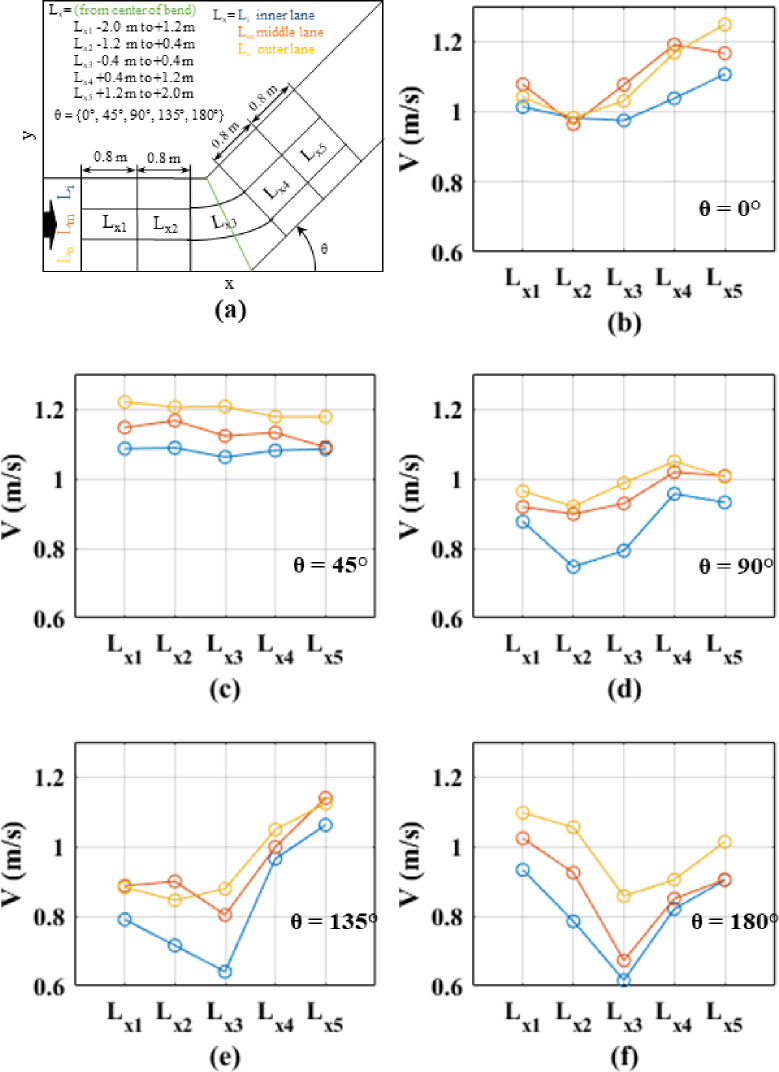
Comparison of lateral and longitudinal speeds along the corridors for normal speed walking; (a) definition of the lanes and cross-sections; (b) straight corridor (0°); (c) 45°; (d) 90°; (e) 135°; (f) 180°.

**Fig 9:**
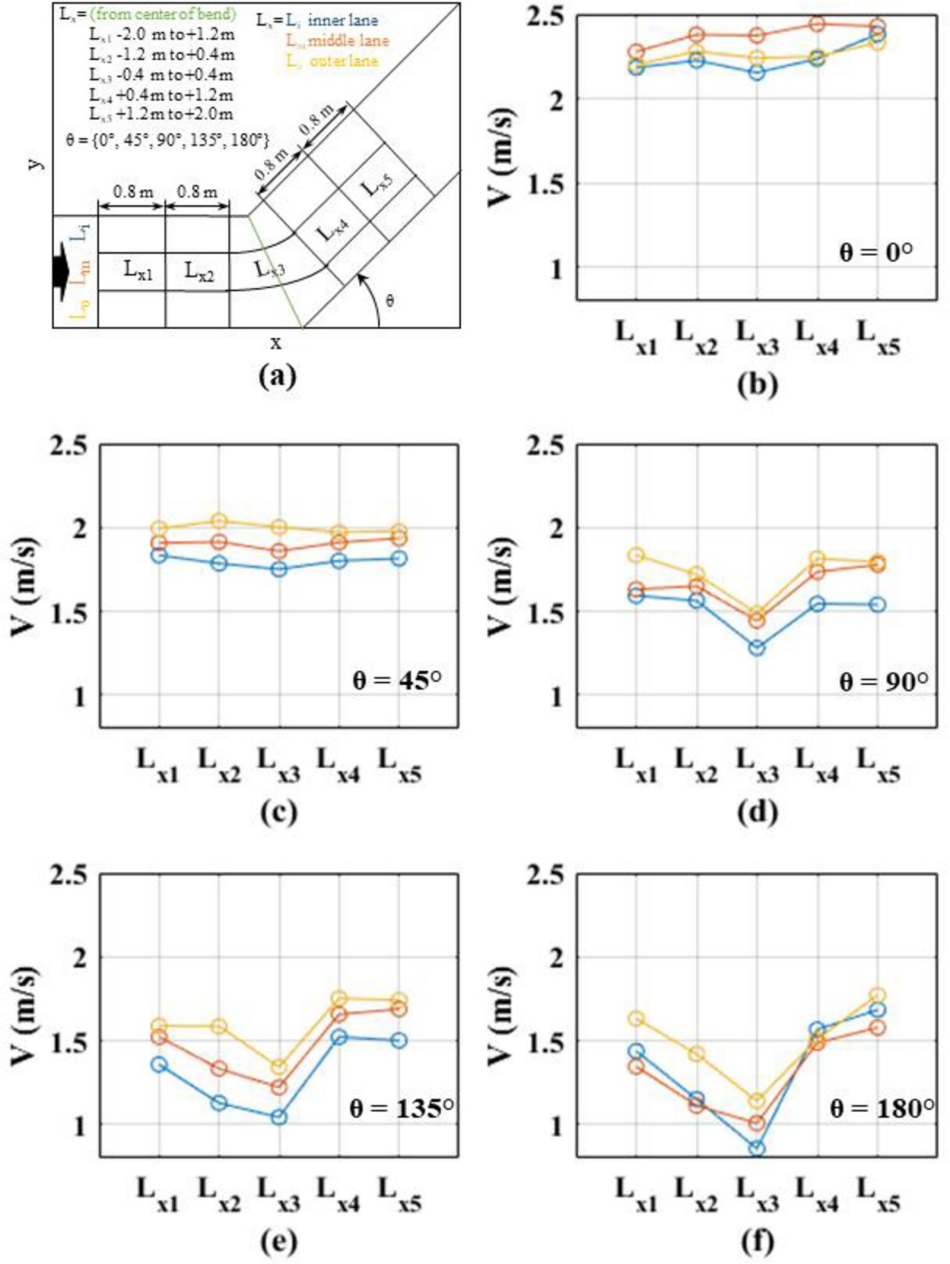
Comparison of lateral and longitudinal speeds along the corridors for slow speed running; (a) definition of the lanes and cross-sections; (b) straight corridor (0°); (c) 45°; (d) 90°; (e) 135°; (f) 180°.

In order to further examine the spatial distribution of speeds, the corridor was divided into several segments as mentioned in Section 3.2. The 1.5 m corridor was divided laterally into 0.5 m lanes to represent inner, middle and outer lanes. Further, a 4 m segment closer to the bend (2 m before and after the inner corner of the bend) was considered by dividing that into five 0.8 m long sub-sections (see Figs 8(A) and 9(A)). That is, altogether 15 segments (3 lateral sections x 5 longitudinal sections) near the bend of the corridor were considered. Instantaneous speeds within these sections were averaged for each turning angle and each speed level to obtain a representative average speed value. These average speeds for different segments within different angled corridors for normal speed walking and jogging cases are compared in Figs 8 and 9, respectively.

Observing the average speeds in Figs 8 and 9, it can be noted that, in general, the speed is lower at L_x3_ region, which is the middle of the corridor. Speed reduction in the middle of the bend is clearly visible for higher angle cases (angles greater than 90°). Comparing average speeds for inner, middle and outer lanes, it can be clearly seen that, for all angles and speed levels, the speeds are lower in the inner lanes compared to outer lanes. Also, when comparing the speed profiles for normal and jogging velocities for the same turning angle, it is noted that the jogging cases display a wider range of speed variations, i.e., lower speeds at the corner and higher speeds at approaching and receding portions of the corridor, can be observed. This observation indicates that the acceleration and deceleration are higher for jogging cases as compared to normal speed walking cases. Further, this effect is magnified when the turning angle is increasing.

One-way ANOVA tests were performed to compare the average speeds within inner, middle, and outer lanes at the bend (i.e., an area within 0.4 m before and after the inner and outer corners) for different angles and speed levels. The outcomes are summarized in Table 3. It can be noted that for straight corridors, average speeds at different lanes (in the middle of the bend) are not statistically different for both speed cases. For the other angles, the differences between the speeds in different lanes (at the bend) were found to be statistically significant. These statistical tests and finding related to Figs 8 and 9 indicate that the speed reduction mainly occurs at the inner corner at the bends. Further, it can be stated that the stampedes could occur even at the inner corner of the smaller turning angles (e.g., 45°), particularly when the walking speeds are higher. A previous study verified that the bends of 45° or more could significantly reduce flow rates under panic conditions [51]. However, according to the same study, the angle threshold was found to be 60° for normal speed walking.

**Table 3:** ANOVA test outcomes for the comparison of average speeds in inner, middle and outer lanes at the bend for different angled corridors.

Scenario		ANOVA test results (F, p-value)
Straight (0°)	Walking	(0.278, 0.757)
	Jogging	(1.902, 0,153)
45°	Walking	(6.964, 0.001) *
	Jogging	(4.303, 0.015) *
90°	Walking	(30.024, 0.000) *
	Jogging	(5.188, 0.006) *
135°	Walking	(50.227, 0.000) *
	Jogging	(17.768, 0.000) *
180°	Walking	(4.667, 0.009) *
	Jogging	(23.473, 0.000) *

* Significant at 0.05 level.

## 5. Discussion and conclusions

It is important to investigate the movements of pedestrians through different corridor layouts under different conditions, e.g., different density and sped levels, in order to enhance corridor designs to avoid bottlenecks due to crowd congestion that may result in stampedes. In particular, evaluating the distribution of the velocities and densities throughout the turning corridors, particularly near the bend area, is important in characterizing and illustrating the bottleneck effect of the corridors with bends. This study used trajectory data of 55 people who traversed different angled corridors, i.e., 0°, 45°, 90°, 135°, and 180°, of 1.5 m wide at two different speed levels, i.e., normal speed walking and slow speed running (jogging) under controlled conditions. Instantaneous velocities (magnitudes and directions) and occupancies were estimated over the walking corridors based on trajectory data obtained from the videos. Using these data, spatial distributions of speeds, walking directions and densities were calculated. Analysis of spatial density distributions explained that corridors with higher angled bends (particularly, 90° or more) could remarkably deteriorate the overall level of service of corridors under normal walking conditions. Pedestrian densities at the bend tend to increase with the increasing turning angle and desired speed. These observations indicate that the bends could become bottlenecks, especially when the turning angle and the desired speeds are high. The variation in densities was higher for larger turning angles under jogging cases. That is, a sudden increase in density (as a result of the sudden decrease in speed) was observed for larger bends at higher speeds. This observation suggests that the bends can trigger the bottleneck effect and could cause stampedes under emergency evacuations, i.e., when the desired speed levels are higher. Several previous studies also verified that the delays due to clogging occurred when people try to move faster, which is a phenomenon called “the faster-is-slower effect” [52–54]. However, recent studies, e.g., Shahhoseini et al. [29], Dias et al. [47], specified that the pedestrian flows or collective discharge could be higher when individuals’ desired speeds are higher. Even though the exit flows could be higher and as a result, the overall performance is enhanced with increasing desired speed, a sudden decrease in speed (and as a result, the sudden increase in densities) at bends could trigger stampedes and trampling. Therefore, turning configurations with higher angles should be avoided at crowd gathering places as much as possible. Previous studies on crowd disasters highlighted that sudden transition of flow conditions, e.g., from laminar to turbulent (stop-and-go conditions), could trigger stampedes resulting in crowd quakes where the sudden release of pressure occurs [55].

Analysis of average speeds at the bend for different angles and speed levels indicated that turning angles below 45° might not decrease the speeds significantly (compared to the straight corridors of the same width). However, the examination of velocity vector distributions and lane-based speed profiles along the turning corridors revealed that walking speeds near the inner corner at the bend are significantly lower compared to the speeds at the middle and outer corners even for smaller turning angles like 45°. These observations indicate that the congestion effect is not uniform over the bend and the bottleneck occurs mainly at the inner corner of the bend. As illustrated in Lee and Hughes [56], a key reason behind the trampling incident that occurred during the Akashi fireworks accident in 2001 in Kobe city, Japan was the turning maneuvers near the staircase and the location of the trampling accident was near the inner corner of the bend. The inner corner of a bend could generally be considered as the shortest path when a group of people negotiates a bend, however, it is not the quickest path [57]. For a given turning angle, people near the inner corner of the bend traverse paths with smaller radii compared to the walkers near the outer corner. Therefore, to enhance the efficiency, design solutions that decrease the sharpness of the inner corner or increase the radius of the walking path could be preferred. In addition, instead of one sharp (or larger) turning angle a series of smaller angles could be placed when designing corners. Such design solutions will aid in mitigating the risk of stampede accidents and enhancing the flow efficiency for the corridors with bends.

Ye et al. [33] investigated both unidirectional and bidirectional flows at a 90° bend. In general, their findings, i.e., that pedestrians walking in the outer lanes have higher velocities, are consistent with the findings of this paper for unidirectional cases. However, because of the interactions between encountering flows, bidirectional flows through turning configurations could be more critical than unidirectional cases. Outcomes of this study could be useful in understanding pedestrian crowd dynamics associated with corridors with bends and exploring potential design solutions. That is, for example, instead of designing corridors with a higher angle, a series of smaller angles could be considered or instead of a sharp corner a smoother, e.g., a curved path, can be set, when designing bends at public crowd gathering places. In addition, the data and findings, particularly related to lane-based speed profiles and spatial distribution of velocity vectors, could be used to develop, calibrate, and validate pedestrian microscopic simulation models to represent realistic pedestrian behaviors through turning configurations. Dias and Lovreglio [58] calibrated cellular automata models to better represent the pedestrian behavior in such models. In the discrete model representation, they divided the turning region into three divisions to distinguish the different behaviors near the corner, in the upstream and the downstream of the bend. The data and findings of this paper may be useful in validating such models and developing models for other angles.

In this study, mainly two parameters, i.e., the turning angle of the bend and the desired speed, were considered. There are other dimensions, e.g., the width of the corridor and initial density, which could influence the overall capacity and the local bottleneck effects of the corridors with bends as well as other geometrical settings. Furthermore, people’s gender, age, and travel purpose may influence their behavior in real-life scenarios. Future studies may include such additional parameters to examine the influence of the complex geometrical layouts on collective pedestrian behaviors.

## Supporting information

S1 Appendix (DOCX)
