# Supplementary material for: Pedestrian flow characteristics through different angled bends: Exploring the spatial variation of velocity
Source: PLoS One. 2022 Mar 3;17(3):e0264635. doi: 10.1371/journal.pone.0264635 (PMC8893709; doi:10.1371/journal.pone.0264635)
Supplement: S1 Appendix — (DOCX) [file pone.0264635.s001.docx]

# **S1 Appendix**

Extracted trajectories are shown in the following figures.


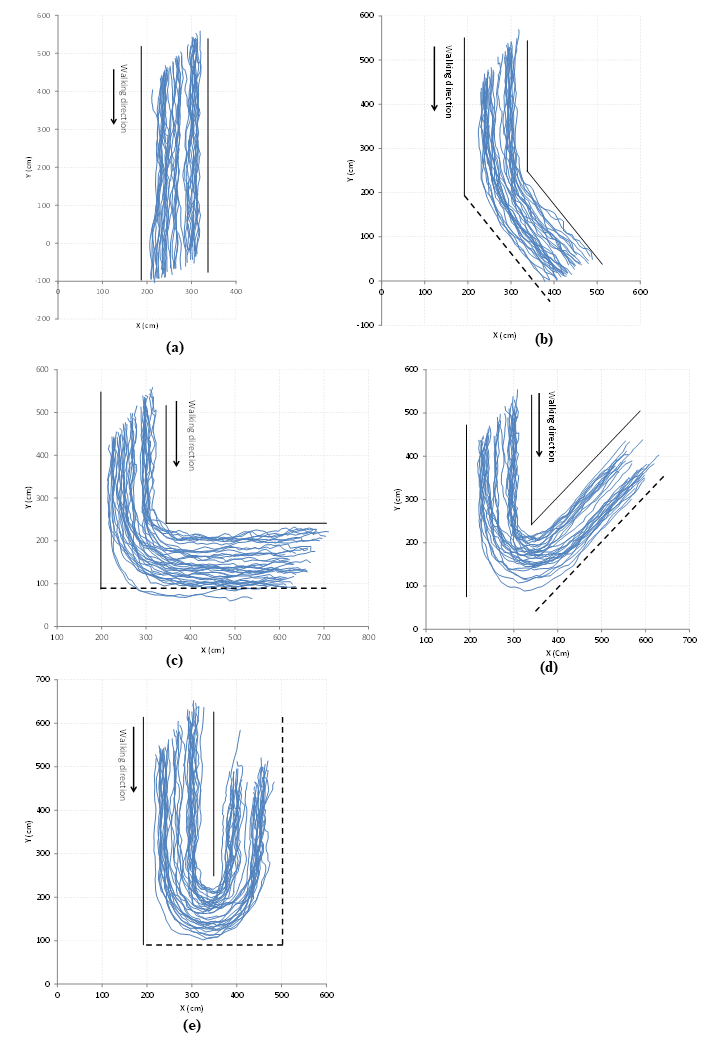


**Fig A-1. Trajectories for normal sped walking cases: (a) 0°; (c) 45°; (d) 90°; (e) 135°; (f) 180°**


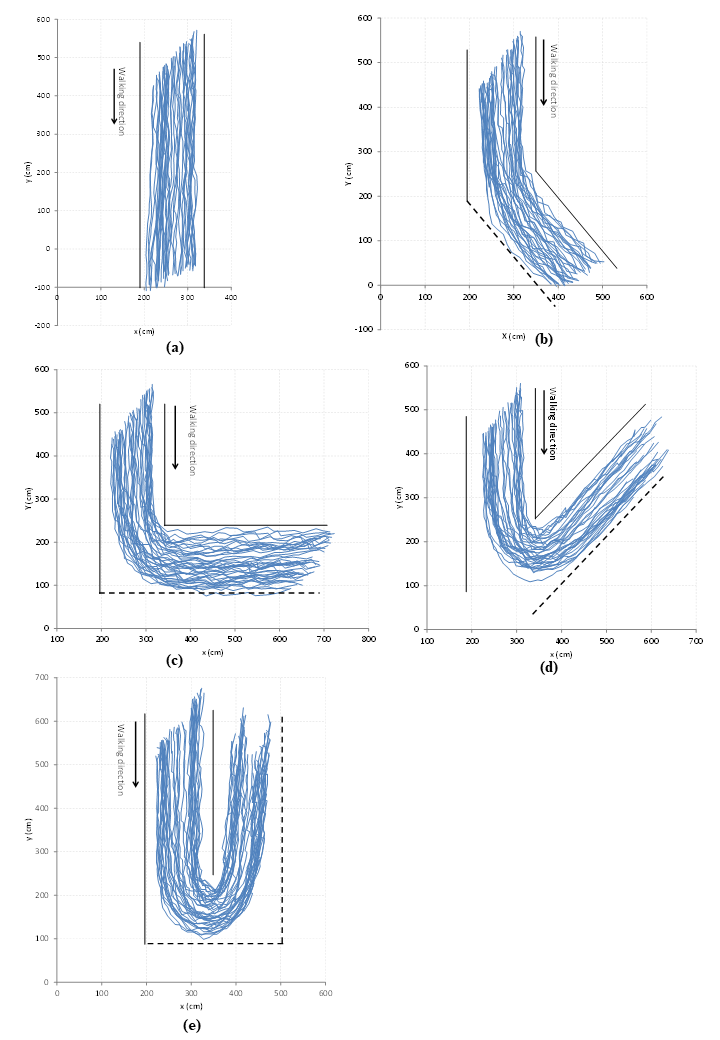


**Fig A-2. Trajectories for jogging cases: (a) 0°; (c) 45°; (d) 90°; (e) 135°; (f) 180°**
